# Activation of Estrogen Receptor β in the Lateral Habenula Improves Ovariectomy-Induced Anxiety-Like Behavior in Rats

**DOI:** 10.3389/fnbeh.2022.817859

**Published:** 2022-05-09

**Authors:** Xiaofeng Liu, Meiying Song, Xiaowei Chen, Yanfei Sun, Renfei Fan, Liping Wang, Weihong Lin, Zheng Hu, Hua Zhao

**Affiliations:** ^1^Key Laboratory of Organ Regeneration and Transplantation of Ministry of Education, Neuroscience Research Center, The First Hospital of Jilin University, Changchun, China; ^2^Department of Physiology, College of Basic Medical Sciences, Jilin University, Changchun, China; ^3^National-Local Joint Engineering Laboratory of Animal Models for Human Diseases, Changchun, China

**Keywords:** ovariectomy, estrogen, anxiety-like behavior, lateral habenula, c-Fos, estrogen receptor beta, monoamine neurotransmitters

## Abstract

**Background:**

Loss of estrogen due to menopause or ovarian resection is involved in the development of anxiety, which negatively impacts work productivity and quality of life. Estrogen modulates mood by binding to estrogen receptors in the brain. Estrogen receptor beta (ERβ) is highly expressed in the lateral habenula (LHb), a key site for controlling the activities of dopaminergic neurons in the ventral tegmental area (VTA) and serotoninergic neurons in the dorsal raphe nucleus (DRN) that are known to be involved in anxiety.

**Methods:**

In this study, we examined the role of LHb in the anxiolytic-like effect of estrogen in ovariectomized (OVX) rats. The establishment of OVX anxiety model was validated in behavioral tests, including elevated plus maze (EPM) and mirror chamber maze (MCM) tasks. The expression of c-Fos in the LHb neurons was analyzed by immunohistochemistry, and monoamine neurotransmitter levels in related nuclei were analyzed using high-performance liquid chromatography (HPLC).

**Results:**

Estrogen-treated OVX rats showed a lower degree of anxiety-like behavior than OVX rats. OVX rats showed anxiety-like behavior and low monoamine levels in the DRN and VTA compared with sham operated and estrogen-treated OVX rats. c-Fos expression in the LHb was higher than that in the sham operated and estrogen-treated OVX rats. Intra-LHb injection of the ERβ-selective agonist diarylprepionitrile (DPN) reduced expression of c-Fos (a neuronal activity marker) and anxiety-like behavior in OVX rats, but not in normal rats, as evidenced by increased time spent in EPM open areas and the MCM mirror chamber. These changes coincided with higher levels of serotonin and dopamine in the DRN and higher dopamine levels in the VTA in OVX rats receiving intra-LHb DPN compared with those receiving vehicle injection.

**Conclusion:**

These results suggest that OVX-induced anxiety-like behavior may be associated with increased LHb activity. DPN may inhibit LHb activity to improve anxiety-like behavior in OVX rats by increasing monoamine neurotransmitter levels in the DRN and VTA.

## Introduction

Loss of estrogen during menopause or consequent ovarian resection can lead to endocrine disorders and neuropsychiatric symptoms, such as memory impairment, insomnia, anxiety, and depression symptoms (Schiller et al., 2016; Soares, 2019; Sovijit et al., 2019). Indeed, 38% of menopausal women reported experiencing anxiety-like symptoms and 36% reported experiencing depressive symptoms (Heidari et al., 2017). Women suffer from anxiety almost twice as much as men (Craske and Stein, 2016). Menopausal women generally have lower levels of monoamine neurotransmitters (Zárate et al., 2002), which may explain why medication (selective serotonin-reuptake inhibitors and serotonin–norepinephrine reuptake inhibitors) for anxiety disorders are effective in the treatment of menopause anxiety (Pinkerton, 2020). These disorders have a profoundly negative impact on quality of life (Craske and Stein, 2016), and it is of great clinical significance to investigate how altered levels of estrogen, in particular estradiol (E2), influence the pathogenesis of neuropsychiatric disorders. Such information will help develop specific therapies for menopausal anxiety disorder.

Studies have shown that anxiety-related behavior is associated with reduced levels of monoamine neurotransmitters, including serotonin (5-HT) and dopamine (DA), and the synthesis and metabolism of these neurotransmitters are influenced by estrogen. For example, ovariectomy-induced anxiety in rats is associated with monoamine levels (Pandaranandaka et al., 2006), and estrogen receptor beta (ERβ) knockout mice exhibit lower monoamine levels in the brain and anxiety symptoms (Krezel et al., 2001; Imwalle et al., 2005). Thus, monoamine neurotransmitters may play an important role in linking anxiety-like behaviors to the loss of estrogen signaling.

The lateral habenula (LHb), located in the epithalamus, is one of the few brain regions that control both dopaminergic and serotonergic systems, and has attracted great attention due to its critical role in regulating mood disorders (Hu et al., 2020). Habenular connectivity with the monoaminergic nervous system may predict treatment responses in depressive psychosis (Gosnell et al., 2019). The dorsal raphe nucleus (DRN) and ventral tegmental area (VTA), the key brain regions for the synthesis and release of 5-HT and DA, are controlled by the LHb (Lecourtier and Kelly, 2007; Zhao et al., 2015; Metzger et al., 2017, 2021). The LHb projects to dopaminergic neurons in the VTA and serotonergic neurons in the DRN (Lecourtier and Kelly, 2007; Baker et al., 2016) and excitation of the LHb inhibits firing of dopaminergic neurons in the VTA (Christoph et al., 1986) and serotonergic neurons in the DRN (Stern et al., 1979).

Anxiety-like behavior in postpartum mice, which is generated by removal of their pups, has been shown to be associated with increased Fos-immunoreactive cell counts in the LHb, suggesting an association between LHb activation and maternal anxiety-like behavior (Smith and Lonstein, 2008). Similarly, increased *c-fos* mRNA expression has been found in the habenula of anxious larval zebrafish (Chen et al., 2015). We previously reported that ovariectomized (OVX) rats had significantly increased *c-fos* mRNA and protein levels in the LHb, and elevated T-type calcium currents in the LHb (Li et al., 2015; Song et al., 2018). Together, these studies suggest that LHb neurons may be activated in an estrogen reduction-induced anxiety state.

Although enrichment of ERβ-expressing cells is found in the LHb (Shughrue et al., 1997; Mitra et al., 2003; Milner et al., 2010) but not in the medial habenula (Simerly et al., 1990; Wagner et al., 1998), the LHb has not been identified as a target of estrogen. In this study, using an OVX rat model, we tested whether estrogen exerts anti-anxiety effects by impacting the LHb and explored the underlying mechanisms involved. Anxiety behavior was assessed using an elevated plus maze (EPM) and a mirror chamber maze (MCM), and neuronal activity was assessed by immunohistochemistry. Monoamine neurotransmitters in the DRN and VTA were determined using an electrochemical high-performance liquid chromatography (HPLC) method to investigate this mechanism.

## Materials and Methods

### Ethics Statement

All animal procedures were carried out in compliance with international ethical guidelines based on the National Institutes of Health Guide for the Care and Use of Laboratory Animals (Council, 2011) and the Management and Use of Experimental Animals published by the National Science and Technology Foundation of China (He et al., 2016). The study was approved by the Committee on the Ethics of Animal Experiments of the First Hospital of Jilin University (ethical code: 2019024). Experimental rats were decapitated under anesthesia for brain sampling, and every effort was made to minimize the pain experienced by the animals.

### Animals

Female Wistar rats (200–220 g) were purchased from Changchun Yisi Experimental Animal Technology Co., Ltd. The 105 animals were divided into different groups. The rats were housed (four rats per cage in the same experimental group) under controlled temperature, humidity, and light conditions with *ad libitum* access to food and water. The lights were programmed on a 12-h light/dark cycle (lights on at 7:00 a.m.). The rats were randomly divided into three groups: non-OVX rats (Sham + Oil group), OVX group (OVX + Oil group), and estrogen supplementation group (OVX + E2 group). Animals with intra-LHb injection of diarylprepionitrile (DPN) were randomly divided into four groups: non-OVX rats and OVX rats were injected with DPN in the LHb (Sham + DPN/OVX + DPN group) or an artificial cerebrospinal fluid (Sham + CSF/OVX + CFS) vehicle. The cannula administration groups were single-caged after cannula installation to allow a full recovery environment and to prevent the destruction of cannula from affecting subsequent experiments. The rats were subjected to fasting and water deprivation for 12 h prior to surgery. Animals were handled daily for at least 3 days prior to behavioral testing to reduce their stress during behavioral testing.

### Drug and Microinjection

17β-Estradiol (E2) (180 μg/ml, E8875) was dissolved in sesame oil (S3547). E2 dosage and administration schedule were based on previous studies (Strom et al., 2008). The ERβ-selective agonist DPN (H5915) was dissolved in dimethyl sulfoxide (DMOS; D2650) as the stock solution and diluted in artificial CSF as a treatment solution (2 μg DPN/μl) (Walf and Frye, 2007; Bastos et al., 2015) to meet the physiological osmotic pressure requirements for brain injection. Rats were injected with the DPN treatment solution (DPN groups) or an equivalent dose of DMSO artificial CSF vehicle (CFS groups). All reagents were purchased from Sigma–Aldrich.

The rats were gently wrapped in a towel during drug microinjection. A dual injection tube connected with a polyethylene tubing (outer diameter (OD), 0.85 mm/inner diameter (ID), 0.42 mm) filled with solution was inserted into the dual guide cannula (OD, 0.48 mm/ID, 0.34 mm). Each polyethylene tube was connected to a 1-μl microsyringe and the solution was injected into the LHb (200 nl/side) using a syringe pump (LSP02-1B, Longer Precision Pump Co., Ltd., China). The needle was left in place for 1 min before injection. The injection lasted for 1 min, and the needle was left in the injection position for 1 min in order to fully diffuse the solution. Behavioral tests were conducted 10 min after the DPN injection (Campos et al., 2018).

### Surgery Procedures

Rats were anesthetized with pentobarbital (intraperitoneal injection, 50 mg/kg). Body temperature was maintained using a thermostatically controlled electric heating pad. Bilateral ovariectomy was performed as previously described (Deurveilher et al., 2009) with minor modifications. OVX rats were administered with E2 to maintain normal E2 stimulation (Li et al., 2015). Under anesthesia, a longitudinal incision was made along the dorsal midline. The muscle layer was drawn from the abdomen through an incision using sterilized hemostatic forceps. The oviducts were clamped using hemostatic forceps and the ovaries were removed. The muscle layer was closed, and then silicone rubber capsules containing E2 dissolved in sesame oil (Dow Corning, United States, 1.57 mm inner diameter × 3.18 mm outer diameter; 20 mm in length), which had been sealed with Type A biogum and pre-incubated in sesame oil for 16 h, were implanted subcutaneously through the incision. Plain sesame oil capsules were implanted under the abdominal skin of the control rats. After surgery, the rats were returned to the housing facilities for 4 weeks until they were evaluated in the behavioral tests. The animals received intraperitoneal injections of 40,000 units of penicillin daily for 3 days.

### Stereotaxic Surgery

Injection sites were determined using a rat brain atlas (Paxinos and Watson, 2007) and verified in a pre-experiment. The LHb injection site coordinates were: –3.5 mm (posterior to bregma), ± 0.7 mm (lateral of bregma), and 4.8 mm (ventral to bregma).

After anesthesia with pentobarbital (intraperitoneal injection, 50 mg/kg), when the rats showed no movement reaction to a foot pinch and had a stable breath rate, they were fixed in a stereotaxic instrument with a rat adaptor (RWD Life Science Co., Ltd., Shenzhen, China), with the head being held still with ear bars. The body temperature was maintained using a thermostatically controlled electric heating pad. The hair on top of the head was shaved with an infant hair clipper and the scalp skin was sterilized with povidone-iodine solution. The scalp was opened with a midline incision and the underlying skull was cleaned with saline to create a clear field. The adapter was adjusted to place the skull at the level position, and holes (diameter 0.6 mm) were drilled with a cranial drill. Dual guide cannula (OD 0.48 mm, ID 0.34 mm, RWD Life Science Co., Ltd., Shenzhen, China) were implanted aiming at the LHb (coordinates above), but the cannula tip was placed lowered to 4.6 mm ventral to the skull surface instead of 4.8 mm leaving space for the injector needle (OD 0.30 mm, ID 0.14 mm, RWD Life Science Co., Ltd., Shenzhen, China) to extend 0.2 mm beyond the tip of the guide cannula. The guide cannula was fixed to the skull surface by using screws and dental cement. The animals received daily intraperitoneal injections of 40,000 units of penicillin for 3 days to prevent infection. The location of the agonist or CFS injected into the LHb was identified by tracing the tip of inserted cannula. The cannula was inserted above the LHb and the inner tube was inserted into the LHb for the injection.

### Immunohistochemistry

The rats were anesthetized with pentobarbital (intraperitoneal injection, 50 mg/kg), and their brains were isolated quickly and immersed in 10% formalin solution for 24 h, but for no more than a week. According to the position of the LHb, the brain was trimmed to contain the LHb and transected to a thickness of 3 mm. The brains were dehydrated, embedded in paraffin, and cut into 5 μm sections using a Leica microtome (RM2245). The sections were deparaffinized with xylene and rehydrated with gradient alcohol, and then treated with sodium citrate at 95°C for 15 min of antigen repair and washed with phosphate-buffered saline (PBS, 0.01 M) for three times. An immunehistochemical kit was used according to the manufacturer’s protocol (MX, Fuzhou, China; catalog no: KIT- 9710). Sections were then incubated with anti-c-Fos (1:1,000, Abcam, Cambridge, United Kingdom, catalog no: ab190289) at 4°C overnight. After completing the staining steps according to the kit instructions, the sections were placed in a color-developing agent (MXB; Fuzhou, China; catalog no: DAB-0031) and the color reaction was terminated with 0.01 M PBS when the color reached an appropriate saturation, and the sections were sealed with neutral resin. The sections were examined under a light microscope (Olympus IX71), and c-Fos immunopositive neurons were counted as an index of neuronal excitability.

### Behavior

The experimental rats were subjected to behavioral tests in numbered order, regardless of the group. Behavioral experiments were performed between 5:00 and 7:00 p.m.

#### Elevated Plus Maze

The EPM is a behavioral test used to assess anxiety in rodents by exploiting the conflict between their natural urge to explore and their aversion to an elevated open environment (Montgomery, 1955; Handley and Mithani, 1984). Our plus-maze apparatus was a cross-shaped black iron plate 70 cm above the ground, consisting of two pairs of opposite-facing arms at right angles to each other. Both arms in one opposite-facing pair were enclosed by 22.5-cm-high iron plate walls with a low light level (10 lx). The other two sides were not enclosed by walls (open arms) and were exposed to moderately bright light (140 lx). Each arm was 15 cm wide and 42.5 cm long, excluding the center 15 cm × 15 cm intersection region of the plus maze.

During the EPM experiment, rats were placed in the middle of the arm intersection. Each animal was monitored for 5 min. A trial was considered invalid if the rat left the maze. The time spent in the maze and the number of entries into the open arms were used as anxiety index parameters. All parameters were measured using a video tracker (EthoVision XT, Noldus, Netherlands). The anxiety index was calculated as follows: 1 - [(open arm time/test duration) + (open arm entries/total entries)]/2. Anxiety index values range from 0 to 1, with a higher anxiety index indicating greater anxiety (Cohen et al., 2008a,b; Mazor et al., 2009). After each trial, the maze was cleaned thoroughly with 75% alcohol to prevent smell interference between the trials.

#### Mirror Chamber Maze

A mirror chamber was used to assess anxious behavior based on the idea that conflict avoidance behaviors are triggered in animals when they see their reflections. The MCM apparatus, which consisted of an open field chamber (76 cm × 57 cm × 35 cm) with four mirrored walls connected to a non-mirrored alleyway (57 cm × 12.5 cm × 35 cm) (Walf et al., 2009), is a modified version of the original mouse apparatus (Toubas et al., 1990).

The rats were placed individually in a mirror chamber, and their movements were recorded for 5 min. A shorter duration of time spent in the mirrored part of the chamber was considered indicative of anxiety. A central area was drawn between the mirror and alley to allow the rats to choose whether to enter the mirror or alley. The anxiety index (modified from our EPM anxiety index) was calculated as 1 –[(Time in the mirror chamber/test duration) + (entries in the mirror chamber/total entries)]/2. The anxiety index values ranged from 0 to 1, with a higher anxiety index indicating greater anxiety. After each trial, the maze was cleaned thoroughly with 75% alcohol to prevent smell interference between the trials.

### High-Performance Liquid Chromatography

Rats were decapitated under deep anesthesia (pentobarbital, 50 mg/kg, intraperitoneal injection) for brain sampling. For the acquisition of brain tissues, rats were anesthetized and decapitated 10 min after DPN or CSF was injected into the LHb. The brain tissue sample needed for separation in the cerebrospinal fluid on ice was stored in a –80°C freezer for later use. Monoamine neurotransmitter concentrations were determined using reverse-phase HPLC coupled with an electrochemical detector (Waters 2,465; Waters Corporation, United States). Each brain tissue sample was weighed, placed in 0.1 M perchloric acid, and then homogenized in a pre-sterilized pestle (PES-15-B-SI, Axygen). The homogenates were subjected to ultrasonication for 10 s (cycle 0.5, amplitude 40%), placed on ice for 30 min, and centrifuged at 12,000 rpm for 15 min at 4°C. Supernatants were filtered through a membrane (0.22 μm) and used immediately for neurotransmitter measurement or stored at –80°C for later use. The monoamine neurotransmitters 5-HT and DA were separated on a Sunfire^®^ C18 column, with a mobile phase containing 120 mM disodium hydrogen orthophosphate (pH 3.2), 250 mg/L sodium octane sulfonate, 80 mg/L EDTA-2Na, 2 mM KCl, and 16% (v/v) methanol. Standard curves were used to quantify the neurotransmitter levels in each sample by calculating the area under the curve. The measured monoamine neurotransmitter concentrations in the brain homogenate were corrected for tissue weight and expressed as ng/mg.

### Statistical Analysis

Rats were randomly assigned to groups. Analyses were performed in a manner blinded to the treatment assignments in all behavioral experiments. Statistical analysis was performed using Prism 8 (GraphPad Software, San Diego, CA, United States). Values were excluded if the drug delivery sites were outside the LHb (pre-established criteria). Statistical differences were determined using one-way analysis of variance (ANOVA) for more than three groups. Data that failed the normality test were analyzed using the Kruskal-Wallis test. When the results of ANOVAs were significant, Tukey’s multiple-comparison *post-hoc* test was conducted. Results are presented as mean ± standard error (SEM), and group sizes are indicated in the figure legends, with “n” representing the number of animals. Correlations between behavioral variables and neurotransmitters in all experimental groups were investigated using Pearson’s correlation.

## Results

### Estrogen Supplementation Alleviated Ovariectomy-Induced Anxiety-Like Behavior

**FIGURE 1:**
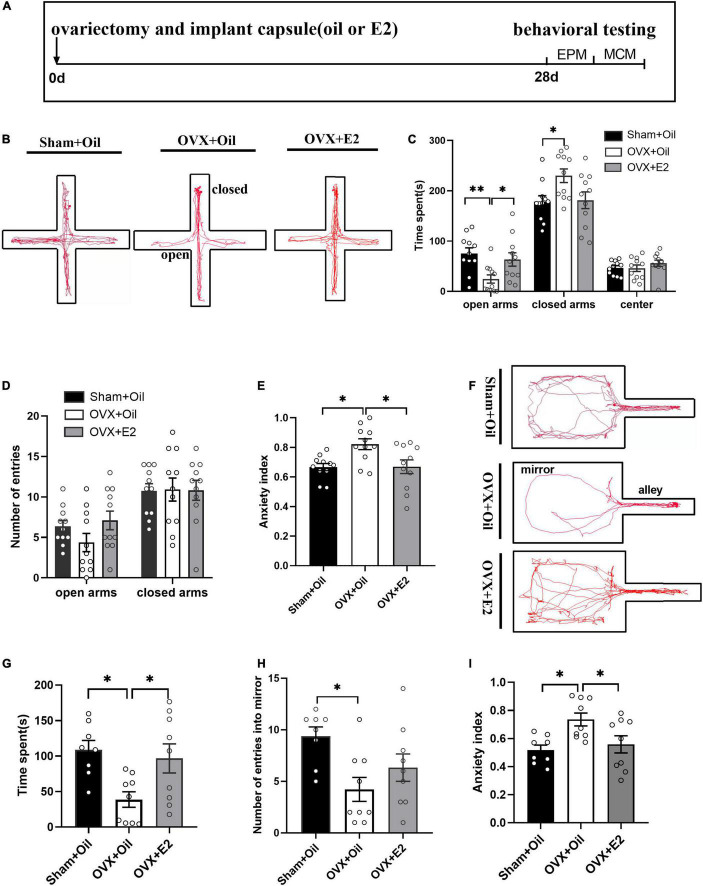
E2 supplementation alleviates anxiety behavior in OVX rats. The timeline of experimental design **(A)**, trajectory maps of EPM **(B)**, times spent in the open arms, closed arms, and center **(C)**, numbers of open-arm entries **(D)**, and mean EPM anxiety index values **(E)** were compared between the Sham + Oil (*n* = 11), OVX + Oil (*n* = 11), and OVX + E2 (*n* = 11) groups during a 5-min EPM test period. The trajectory maps of MCM **(F)**, times spent in the mirror chamber **(G)**, numbers of mirror chamber entries **(H)**, and mean MCM anxiety index values **(I)** were compared among the Sham + Oil (*n* = 8), OVX + Oil (*n* = 9), and OVX + E2 (*n* = 9) groups. *p < 0.05, **p < 0.01.

To study the behavior of rats after ovariectomy, we divided the rats into three groups and tested the elevated plus maze and mirror chamber maze after the different treatments (Figure 1A). Figure 1B showed the movement tracks of the elevated plus maze of rats. In the EPM, the results showed that compared with the Sham + Oil group, OVX + Oil rats spent less time in the open arms (*p* < 0.01; Figure 1C), more time in the closed arms (*p* < 0.05; Figure 1C), and showed a higher anxiety index (*p* < 0.05; Figure 1E). The OVX + E2 group had a lower anxiety index than the OVX + Oil group (*p* < 0.05; Figure 1E), which improved this anxiety state. There was no statistical difference in the number of open and closed arm explorations among the three groups (Figure 1D).

The movement tracks in mirror chamber maze are shown in Figure 1F. Compared to the Sham + Oil group, the OVX + Oil group spent significantly less time exploring the mirror chamber (*p* < 0.05; Figure 1G) and had fewer entries into the mirror chamber (*p* < 0.05; Figure 1H), showing a higher mean anxiety index value (*p* < 0.05; Figure 1I). The OVX + E2 group spent more time exploring the mirror chamber and had a lower anxiety index than the OVX + Oil group, which improved this anxiety state.

### Estrogen Supplementation Alleviated Ovariectomy-Induced the Activation of c-Fos Expression and Changes in Neurotransmitters

**FIGURE 2:**
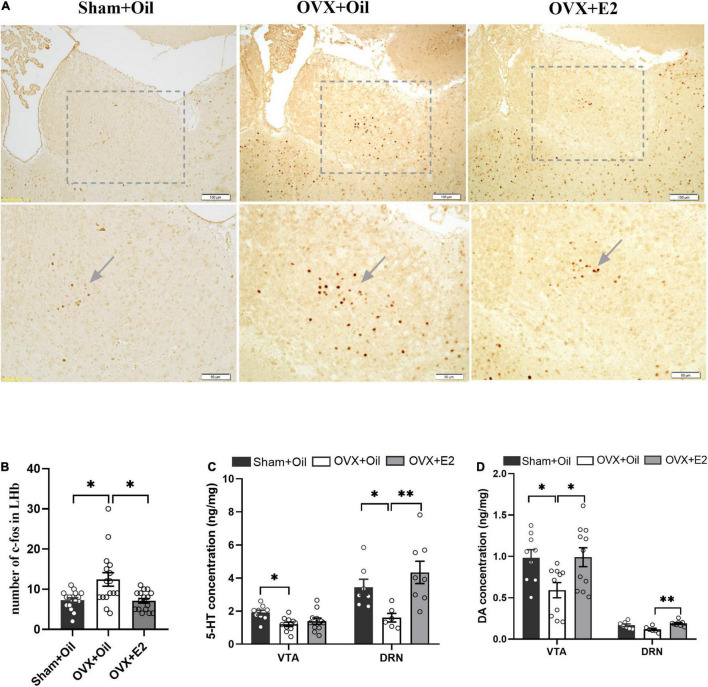
Systemic administration of estrogen reduces c-Fos expression in the LHb and increases the concentrations of monoamine neurotransmitters in the DRN and VTA of OVX rats. The representative images of c-Fos expression in the Sham + Oil, OVX + Oil and OVX + E2 rats **(A)**. Systemic administration of estrogen induced decreased density of c-Fos cells in the LHb. Data are presented as the quantitative analysis of c-Fos positive neurons in the Sham + Oil, OVX + Oil, and OVX + E2 groups **(B)**. Comparison of 5-HT **(C)** and DA **(D)** concentrations in the VTA and DRN among the Sham + Oil (*n* = 9, 7), OVX + Oil (*n* = 10, 6), and OVX + E2 (*n* = 11, 8) groups. *p < 0.05, **p < 0.01.

Immunohistochemistry results showed that the number of c-Fos in the LHb increased significantly in the OVX + Oil group compared to that in the Sham + Oil group (*p* < 0.05, Figures 2A,B). c-Fos expression in the OVX + E2 group was significantly lower than that in the OVX + Oil group (*p* < 0.05, Figures 2A,B).

Results of the HPLC analysis showed that OVX + Oil group had significantly lower levels of 5-HT (*p* < 0.05) and marginally lower DA (*p* = 0.06) in the DRN compared with the Sham + Oil group. The 5-HT and DA levels in the VTA were significantly lower than those in the Sham + Oil group (*p* < 0.05 for 5-HT and DA). Compared with the OVX + Oil group, the OVX + E2 group showed higher levels of 5-HT and DA (*p* < 0.01) in the DRN and DA (*p* < 0.05) in the VTA (Figure 2D), indicating that the reduction in monoamine neurotransmitters may be improved after estrogen supplementation (Figures 2C,D).

### Intra-Lateral Habenula Diarylprepionitrile Attenuated Anxiety-Like Behavior in Ovariectomized Rats

**FIGURE 3:**
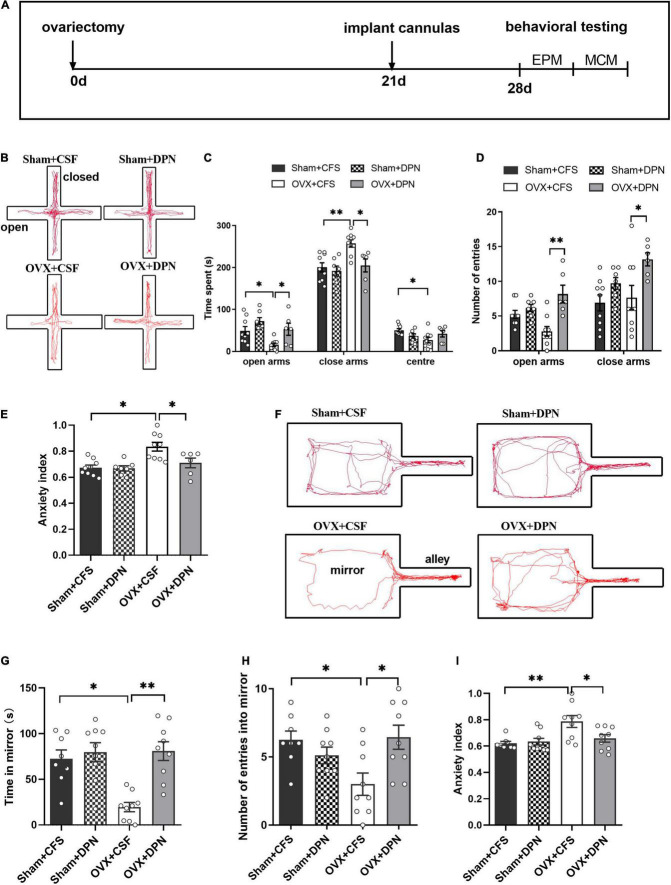
Intra-LHb injection of DPN improves anxiety behavior in sham and OVX rats. Timeline of experimental design **(A)**. The trajectory maps of EPM **(B)**, times spent in the open arms, closed arms, and center **(C)**, numbers of open-arm entries **(D)**, and mean EPM anxiety index values **(E)** were compared among the Sham + CFS (*n* = 9), Sham + DPN (*n* = 7), OVX + CFS (*n* = 9), and OVX + DPN (*n* = 6) groups during a 5-min EPM test. The trajectory maps of MCM **(F)**, times spent in the mirror chamber **(G)**, numbers of mirror chamber entries **(H)**, and mean MCM anxiety index values **(I)** were compared between the Sham + CFS (*n* = 8), Sham + DPN (*n* = 9), OVX + CFS (*n* = 9), and OVX + DPN (*n* = 9) groups. *p < 0.05; **p < 0.01.

To test whether estrogen could improve anxiety-like behavior in OVX rats through the LHb, we investigated behavioral changes in OVX rats by injecting DPN into the LHb. A schematic of the experimental design (Figure 3A), and anxiety behavioral data obtained from the LHb-microinjected OVX rats are shown in Figure 3. In the EPM, compared with OVX + CSF rats, OVX + DPN rats spent significantly more time in the open arms and less time in the closed arms (*p* < 0.05; Figure 3C), had significantly more open arm entries (*p* < 0.01) and closed arm entries (*p* < 0.05; Figure 3D), and had a significantly reduced mean EPM anxiety index value (*p* < 0.05; Figure 3E). Compared with the OVX + CSF rats, the OVX + DPN rats spent significantly more time in the mirror chamber (*p* < 0.01; Figure 3G), showed significantly more entries into the mirror chamber (*p* < 0.05; Figure 3H), and had a significantly reduced mean MCM anxiety index value (*p* < 0.05; Figure 3I). There was no significant difference between the Sham + CFS group and the Sham + DPN group in any behavioral change.

### Intra-Lateral Habenula Injection of Diarylprepionitrile Can Reduce c-Fos Expression in the Lateral Habenula and Increase Monoamine Levels in the Dorsal Raphe Nucleus and Ventral Tegmental Area of Ovariectomized Rats

**FIGURE 4:**
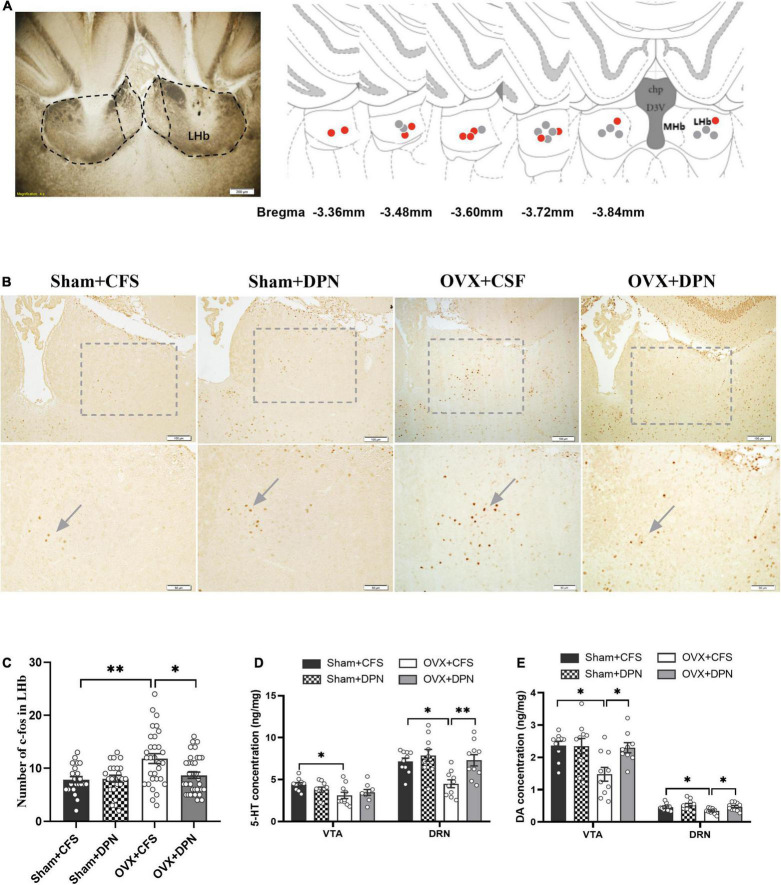
Intra-LHb injection of DPN reduces c-Fos expression in the LHb and increases concentrations of monoamine neurotransmitters in the DRN and VTA of the Sham and OVX rats. Location of the cannula in the LHb (**A**, left). Coronal sections are 3.36–3.84 mm posterior to bregma. Red and gray circles represent injection sites in the LHb of OVX + DPN and OVX + CFS rats in the LHb, respectively (**A**, right). The representative images of c-Fos expression in the Sham + CFS, Sham + DPN, OVX + CSF, and OVX + DPN groups **(B)**. Intra-LHb injection of DPN induced decreased in the density of c-Fos cells in LHb **(C)**. Data are presented as quantitative analysis of c-Fos positive neurons in the LHb. Comparison of 5-HT **(D)** and DA concentrations **(E)** in the VTA and DRN among the Sham + CFS (*n* = 10, 10), Sham + DPN (*n* = 10, 10), OVX + CSF (*n* = 10, 10), and OVX + DPN (*n* = 9, 10) groups. *p < 0.05; **p < 0.01.

The locations for microinjection of DPN and CSF to the LHb in the OVX rats are shown in Figure 4A. We quantitatively evaluated the number of c-Fos positive cells in LHb after DPN was injected to the LHb. Immunohistochemical images suggested that the number of c-Fos cells in the LHb was reduced in response to DPN microinjection into the LHb (Figure 4B). Marked differences were detected in the LHb. There was a decrease in the number of c-Fos in the LHb of rats in the OVX + DPN group when compared to OVX + CFS group (*p* < 0.01; Figure 4C) suggesting that DPN reduced neuronal excitability. There was no change in the number of c-Fos in the LHb following CFS and DPN injections in sham rats.

In the DRN, the 5-HT (*p* < 0.01; Figure 4D) and DA (*p* < 0.05; Figure 4E) concentrations were significantly higher in the OVX + DPN group than in the OVX + CSF group. In the VTA, DA concentrations were significantly higher in the OVX + DPN group than in the OVX + CSF group (*p* < 0.05; Figure 4E). CFS and DPN injections in sham rats did not change the concentrations of DRN or VTA neurotransmitters.

### Correlation Between Behavioral Variables and Neurotransmitters in the Ventral Tegmental Area and Dorsal Raphe Nucleus

We analyzed the correlations between the behavioral variables and the levels of monoamine neurotransmitters in the VTA and DRN of OVX + Oil and OVX + E2 groups (Table 1) and intra-LHb injection of OVX + CFS and OVX + DPN groups (Table 2). In the systemic estrogen experimental groups, Pearson’s correlation coefficient indicated that DA in the VTA and 5-HT in the DRN were significantly and positively correlated with the time spent on the open arms in the EPM and the time/entries in the mirror chamber, but a significant negative correlation was found with the anxiety index in the EPM/MCM and the time spent on the closed arms in the EPM. In the MCM, the DA in the DRN is positively correlated with the exploration into the mirror and negatively correlated with the anxiety index. Correlations similar to those found in the systemic administration groups were also observed in the OVX + CFS and OVX + DPN groups.

**TABLE 1:** Corrections between behavioral variables and neurotransmitters in the VTA and DRN of OVX + Oli and OVX + E2 groups.

	VTA				DRN			
Behavioral variables	DA (ng/mg)		5-HT (ng/mg)		DA (ng/mg)		5-HT (ng/mg)	
	*r*	*p*	R	*p*	*r*	*p*	*r*	*p*
**Elevated plus maze**								
Open arms (s)	0.694	0.006	0.276	0.338	0.117	0.689	0.718	0.004
Closed arm (s)	–0.624	0.017	–0.266	0.358	–0.088	0.762	–0.625	0.017
Entries open arms (*n*)	0.472	0.088	0.258	0.372	–0.164	0.574	0.465	0.093
Entries closed arms (*n*)	–0.192	0.509	0.079	0.788	–0.301	0.295	–0.315	0.272
Anxiety index	–0.690	0.006	–0.232	0.424	–0.097	0.741	–0.738	0.003
**Mirror chamber maze**								
Mirror (s)	0.592	0.026	0.190	0.515	0.548	0.042	0.671	0.009
Entries mirror (*n*)	0.760	0.002	0.468	0.091	0.430	0.124	0.585	0.028
Anxiety index	–0.627	0.016	–0.300	0.297	–0.607	0.021	–0.650	0.012

**TABLE 2:** Corrections between behavioral variables and neurotransmitters in the VTA and DRN of OVX + CFS and OVX + DPN groups.

	VTA				DRN			
Behavioral variables	DA (ng/mg)		5-HT (ng/mg)		DA (ng/mg)		5-HT (ng/mg)	
	*r*	*p*	R	*p*	*r*	*p*	*r*	*p*
**Elevated plus maze**								
Open arms (s)	0.586	0.022	–0.109	0.699	0.188	0.502	0.671	0.006
Closed arm (s)	–0.660	0.007	0.011	0.968	–0.320	0.230	–0.733	0.002
Entries open arms (*n*)	0.590	0.020	0.044	0.876	0.384	0.157	0.668	0.007
Entries closed arms (*n*)	0.414	0.124	–0.121	0.666	0.391	0.146	0.557	0.030
Anxiety index	–0.560	0.030	–0.144	0.607	–0.298	0.281	–0.576	0.024
**Mirror chamber maze**								
Mirror (s)	0.688	0.005	–0.137	0.624	0.491	0.063	0.782	0.001
Entries mirror (*n*)	0.655	0.008	–0.089	0.751	0.592	0.020	0.689	0.004
Anxiety index	–0.670	0.006	–0.093	0.740	–0.541	0.037	–0.647	0.009

*(s), time in seconds; (n), number; r, Pearson’s correlation coefficient; VTA, ventral tegmental area; DRN, dorsal raphe nucleus; DA, dopamine; 5-HT, serotonin.*

## Discussion

It has been reported that the loss of estrogen can induce anxiety behavior, and its pathogenesis involves abnormal function of the estrogen β receptor. The LHb plays an important role in the development of depression and anxiety (Metzger et al., 2021) and has a large distribution of estrogen β receptors (Milner et al., 2010). However, it is still unclear whether LHb mediates the anti-anxiety effects of estrogen.

In the present study, we showed that ovariectomy-induced anxiety behavior could be attenuated with E2 supplementation, as evidenced by improved performance in the exploration of the open spaces of the EPM and the mirror chamber of the MCM. The LHb showed increased c-Fos expression in ovariectomized rats, and these changes were reversed by estrogen supplementation. We further found that intra-LHb injection of the ERβ-selective agonist DPN in OVX rats reduced the number of c-Fos positive cells and increased monoamine neurotransmitter levels in the DRN and VTA, suggesting that reduced neuronal activity of DPN in the LHb may relieve anxiety-like behavior induced by the loss of estrogen, possibly through increasing the levels of monoamine neurotransmitters in the DRN and VTA. The significant correlations between the behavioral variables and the levels of monoamine neurotransmitters in the VTA and DRN in all intra-LHb injections of DPN experimental groups further supports the above hypothesis.

We conducted animal behavioral experiments 4 weeks after ovariectomy in rats. The rats with 4 weeks of ovariectomy exhibited anxious behavior in the EPM and MCM tasks, and estrogen supplementation improved the anxious behavior. These results are consistent with previous reports showing that loss of estrogen (Pandaranandaka et al., 2006) or OVX rats exhibited increased anxiety-like behaviors in the light-dark shuttle box and locomotor activity tests (Fedotova et al., 2017; Puga-Olguín et al., 2019). Thus, we are confident that, in this study, the model was successful, and the results generated from using this model should be reliable.

The LHb in OVX rats showed an increased expression of c-Fos, which was inhibited by estrogen supplementation, which was consistent with our previous results showing increased c-Fos expression in the LHb 1 week after ovariectomy (Li et al., 2015). We previously demonstrated that estrogen supplementation not only reversed the increase in c-Fos expression in the LHb of OVX rats but also reversed the expression of Cav3.3 T-type calcium channels and T-type calcium currents in the LHb of OVX rats (Song et al., 2018), which is critical for regulating neuronal excitability (Arbogast, 2008). More importantly, estradiol suppressed spontaneous firing activity in the LHb neurons compared with firing rates prior to estradiol treatment using whole-cell recording (Song et al., 2018). These results suggest that ovariectomy-induced anxiety behavior may be related to changes in LHb neuronal activity mediated by T-type calcium channels and that the LHb may be a potential target for estradiol action.

In the present study, we found that the ERβ-selective agonist DPN microinjected into the LHb of OVX rats alleviated ovariectomy-induced anxiety behavior, as evidenced by improved performance in the exploration of the open arms of the EPM and the mirror chamber of the MCM. DPN also decreased c-Fos expression in the LHb of OVX rats and increased monoamine neurotransmitter levels in the DRN and the VTA. Estrogen regulates brain function by affecting the release of monoamine neurotransmitters (Pandaranandaka et al., 2006; Long et al., 2018). In the present study, rats showing anxiety-like behavior following ovariectomy had lower levels of 5-HT/DA in the DRN and DA in the VTA. 5-HT and DA, which play important roles in anxiety-like behaviors (Liu et al., 2018; Schanzer et al., 2019), are synthesized and released into the DRN and VTA, respectively. It has been suggested A decrease in dopaminergic neuronal activity in the VTA may be the basis of anxiety-related behavior (Coque et al., 2011; Rincón-Cortés et al., 2018; Rincon-Cortes and Grace, 2020) and the inhibition of 5-HT neurons in the DRN could induce anxiety-related behaviors in mice and rats (Nishitani et al., 2019). Correlation analysis showed that DA in the VTA and 5-HT in the DRN were correlated with behavioral variables in the EPM and MCM in all systemic estrogen experimental groups, which supports the hypothesis that the DRN and VTA are involved in the development of anxiety-like behavior.

The LHb plays an important role in the pathogenesis of anxiety and depression (Yang et al., 2008; Zhao et al., 2015) and is a key site for controlling neuronal activity in the DRN and VTA. Activation of the LHb inhibits neuronal activity in the DRN and VTA. OVX rats exhibited hyperactivity of neurons in the LHb in the present and previous studies (Li et al., 2015). Therefore, we hypothesized that OVX-induced anxiety-like behavior may be attributed to increased neuronal activity of the LHb, leading to reduced neuronal activity of the DRN and VTA, which reduces the release of 5-HT and DA neurotransmitters. DPN microinjected into the LHb of OVX rats suppressed the activity of LHb neurons, as evidenced by decreased c-Fos expression and increased levels of 5-HT and DA in the DRN and DA in the VTA. We suggest that DPN in the LHb suppresses its activity, which weakens the inhibitory effect on the DRN and VTA, leading to elevated DRN and VTA neuronal activity and alleviation of anxiety-like behavior.

Ovariectomy has been reported to reduce gamma-aminobutyric acid (GABA) levels and GABA_*A*_ receptor expression in the hippocampus, amygdala, and other brain structures (Herbison and Fénelon, 1995; Tominaga et al., 2001). Binding of estrogen to receptors can increase the synthesis of the inhibitory neurotransmitter GABA in the brain (McCarthy, 2008). Therefore, we hypothesized that DPN injection into the LHb may activate GABA neurons and thus inhibit the neuronal activity of the LHb neurons, leading to improved anxiety behavior in ovariectomized rats.

It has been reported that c-Fos expression decreases in the lateral septal nucleus of ovariectomized rats and is restored when E2 is administered to ovariectomized rats (Puga-Olguín et al., 2019). About 75% of GABAergic neurons in the septal nucleus project to the LHb and inhibit its activity (Golden et al., 2016). Therefore, we hypothesized that the reduced activity of GABA neurons projected from the lateral septal nucleus to the LHb weakened its inhibitory effect on the LHb leading to increased LHb activity and further inhibition of the activity of 5-HT neurons in the DRN and DA neurons in the VTA, which may be involved in the development of ovariectomy-induced anxiety behavior. There is a morphological and functional connection between VTA and DRN neurons, thus the interaction between them during ovariectomy-induced anxiety behavior needs to be further studied.

In summary, estrogen loss can cause anxiety-related behaviors, and the LHb, which highly expresses ERβ, appears to be associated with the development of anxiety-related behaviors. In this study, we demonstrated the effects of estrogen deficiency induced by ovariectomy in rats, including increased anxiety-like behaviors, as well as decreased levels of 5-HT and DA in the DRN and DA in the VTA. It was further found that systemic estrogen supplementation or local ERβ agonism within the LHb could improve anxiety-like behavior and reverse estrogen-deficiency-induced decreases in monoamine neurotransmitter levels of the DRN and VTA. These results support our hypothesis that the LHb plays an important role in anxiety associated with estrogen deficiency owing to its influence on 5-HT and DA levels in the DRN and DA levels in the VTA.

## Data Availability Statement

The original contributions presented in the study are included in the article/supplementary material, further inquiries can be directed to the corresponding author/s.

## Ethics Statement

The animal study was reviewed and approved by the Ethics of Animal Experiments of the First Hospital of Jilin University.

## Author Contributions

XL conducted the experiments and wrote the manuscript. MS and XC assisted in conducting the experiments. YS, RF, LW, and WL assisted in preparing materials for the experiment. ZH reviewed and edited the manuscript. HZ designed the study, supervised all aspects of the study, and wrote the manuscript. All authors approved the final version of the manuscript.

## Conflict of Interest

The authors declare that the research was conducted in the absence of any commercial or financial relationships that could be construed as a potential conflict of interest.

## Publisher’s Note

All claims expressed in this article are solely those of the authors and do not necessarily represent those of their affiliated organizations, or those of the publisher, the editors and the reviewers. Any product that may be evaluated in this article, or claim that may be made by its manufacturer, is not guaranteed or endorsed by the publisher.
